# Submarine groundwater discharge data at meter scale (^223^Ra, ^224^Ra, ^226^Ra, ^228^Ra and ^222^Rn) in Indian River Bay (Delaware, US)

**DOI:** 10.1016/j.dib.2019.104728

**Published:** 2019-10-28

**Authors:** Carlos Duque, Karen L. Knee, Christopher J. Russoniello, Mahmoud Sherif, Usama A. Abu Risha, Neil C. Sturchio, Holly A. Michael

**Affiliations:** a WATEC, Department of Geoscience, Aarhus University, Aarhus, Denmark; b Department of Geological Sciences, University of Delaware, Newark, DE, USA; c Department of Environmental Science, American University, Washington, DC, USA; d Department of Geology and Geography, West Virginia University, Morgantown, WV, USA; e Department of Geology, Tanta University, Tanta, Egypt; f Geology Department, Desert Research Center, Cairo, Egypt; g Department of Civil and Environmental Engineering, University of Delaware, Newark, DE, USA

**Keywords:** Submarine groundwater discharge (SGD), Radon (Rn), Radium (Ra), Coastal groundwater, Radioactive tracers, Indian River bay, DE, USA

## Abstract

Submarine groundwater discharge (SGD) was sampled at high-spatial resolution in Indian River Bay, DE, USA, in July 2016 to characterize the spatial variability of the activity of the radium and radon isotopes commonly used to estimate SGD. These data were part of an investigation into the methods and challenges of characterizing SGD rates and variability, especially in the coastal aquifer transition from freshwater to saltwater (Hydrogeological processes and near shore spatial variability of radium and radon isotopes for the characterization of submarine groundwater discharge (Duque et al., 2019)). Samples were collected with seepage meters and minipiezometers to obtain sufficient volumes for analytical characterization. Seepage meter samples (for ^223^Ra, ^224^Ra, ^226^Ra, and ^228^Ra) were collected at two-hour intervals over a semi-diurnal tidal cycle from 30 seepage meters. Samples for ^222^Rn characterization were collected with a minipiezometer from 25 cm below the bay bed at each seepage meter location. All samples were analyzed with standard and state of the art procedures.

**Table:** Specifications Table

Subject	Water Science and Technology
Specific subject area	^223^Ra,^224^Ra,^226^Ra,^228^Ra,^222^Rn and specific conductance data collected from submarine groundwater discharge
Type of data	Table
How data were acquired	Submarine groundwater discharge water was collected from Lee-type seepage meters [2] and analyzed for naturally occurring radium (^223^Ra,^224^Ra,^226^Ra,^228^Ra) Minipiezometers collected porewater that was analyzed for naturally occurring radon (^222^Rn) Instruments for analysis: For^223^Ra and^224^Ra: Radium Delayed Coincidence Counter (RaDeCC) For^226^Ra and^228^Ra: High-purity Ge gamma spectrometers (Model GWL-170-15-LB-AWT with 15 mm well diameter, EG&G Ortec, Ametek, Inc.) For^222^Rn: RAD7 radon detector with RAD H2O accessory (Durridge Co., Billerica, MA)
Data format	Raw
Parameters for data collection	The field site was monitored at high spatial resolution for the collection of groundwater directly discharging to a shallow bay
Description of data collection	A grid of 6 × 5 seepage meters was installed with 3 m distance between seepage meters and used to collect samples for measurement of SGD radium activity. Minipiezometers were used to collect porewater samples adjacent to seepage meters to analyse radon activities. All samples were collected over a semi-diurnal tidal cycle.
Data source location	City/Town/Region: Holts Landing State Park Country: US (DE) Latitude and longitude for collected samples. UTM coordinates: 488936.4, 4271469.7
Data accessibility	With the article
Related research article	Duque C., Knee K.L., Russoniello C.J., Sherif M., Abu Risha U.A., Sturchio N.C., Michael H.A., 2019. Hydrogeological processes and near shore spatial variability of radium and radon isotopes for the characterization of submarine groundwater discharge. Journal of Hydrology

**Table:** 

**Value of the Data** • This dataset is unique because of the high spatial-resolution (<3 m), number of samples (n = 30), sampling methods and salinity changes in groundwater. • Samples have been collected directly from submarine groundwater discharge (SGD) through seepage meters or shallow piezometers. • This dataset can be used in comparisons and syntheses about radioactive tracer-based SGD estimates in regional and global studies (^223^Ra,^224^Ra,^226^Ra,^228^Ra,^222^Rn).

## 1 Data

**Fig. 1:**
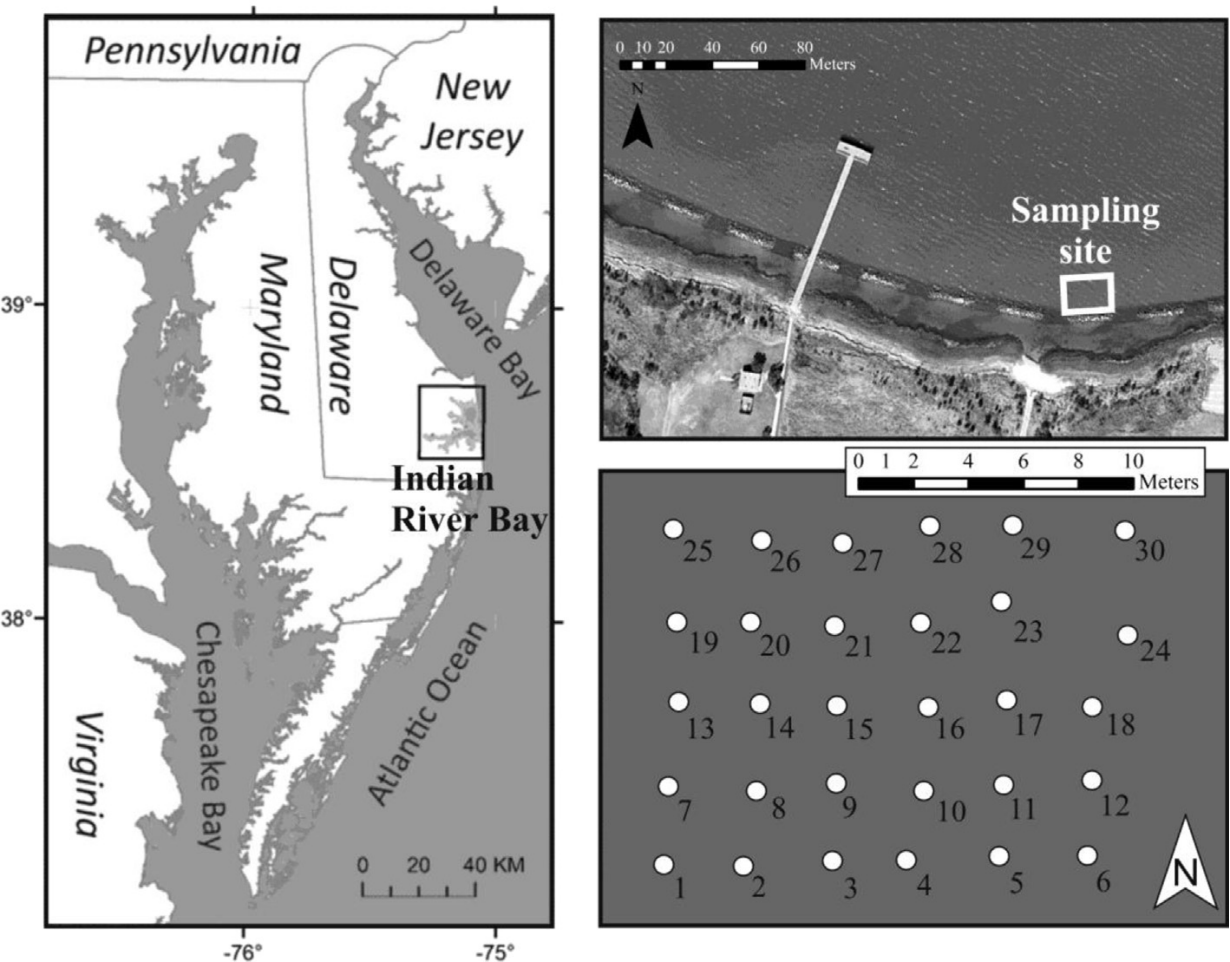
Location of Indian River Bay, sampling site and structure and number of sampling points (UTM coordinates for each location are presented in Table 1) modified from Duque et al. [1].

Table 1 contains the locations, sample volumes, and radioactive tracer activities at each of 30 sampling locations (Fig. 1) where a seepage meter was installed in Indian River Bay (DE) on July 2016. Groundwater was collected from seepage meter bags for later laboratory analysis. A porewater sample from 25 cm depth was also collected during this sampling period near each seepage meter. Sample volumes depended on SGD rates, so collected volumes varied between the 30 locations (Table 1). Activities of ^223^Ra, ^224^Ra, ^226^Ra, ^228^Ra, and ^222^Rn were later determined in the laboratory and are presented, along with the total propagated uncertainty of the analytical methods (Table 1). The specific conductance (SC) is reported for each sample, because it affects Ra activity and also act as a salinity proxy and indicator of the origin of discharging groundwater. A bay water sample, collected in proximity of the study area, is provided for comparison.

**Table 1:** Universal Transverse Mercator (UTM) coordinates, ^223^Ra, ^224^Ra, ^226^Ra, ^228^Ra, ^222^Rn activities with analytical error, volume of sample (V) and specific conductance (SC).

Sample	UTM X	UTM Y	^226^Ra (dpm/100L)	^228^Ra (dpm/100L)	^223^Ra (dpm/100L)	^224^Ra (dpm/100L)	V (L)	^222^Rn (pCi/L)	SC (mS/cm)
S1	488931.1	4271464.0	29 ± 34	5 ± 3	1 ± 1	19 ± 14	15.55	166 ± 12	4.61
S2	488934.0	4271464.0	15 ± 30	7 ± 3	1 ± 0	12 ± 4	20.85	33 ± 5	5.79
S3	488936.7	4271464.1	13 ± 3	2 ± 1	0 ± 0	4 ± 1	46.40	192.3 ± 14	1.28
S4	488939.6	4271464.3	7 ± 2	6 ± 1	0 ± 0	4 ± 2	60.30	62 ± 4	0.97
S5	488942.8	4271464.7	8 ± 15	1 ± 1	0 ± 0	6 ± 1	35.40	71 ± 5	1.76
S6	488945.7	4271464.5	20 ± 5	5 ± 2	0 ± 0	6 ± 2	22.30	49 ± 7	1.74
S7	488931.1	4271466.3	1 ± 15	31 ± 3	3 ± 1	108 ± 9	33.40	95 ± 8	7.39
S8	488933.1	4271466.5	15 ± 4	31 ± 2	2 ± 1	87 ± 7	45.50	165 ± 12	4.93
S9	488936.2	4271466.6	21 ± 4	39 ± 2	1 ± 0	44 ± 5	40.35	137 ± 10	4.76
S10	488939.0	4271466.9	18 ± 3	16 ± 2	0 ± 0	18 ± 5	31.50	74 ± 54	4.54
S11	488942.0	4271466.8	37 ± 2	48 ± 1	2 ± 1	102 ± 8	58.40	65 ± 5	6.85
S12	488945.2	4271466.9	29 ± 3	51 ± 2	1 ± 1	117 ± 7	58.90	24 ± 4	3.89
S13	488930.5	4271469.3	40 ± 11	284 ± 9	39 ± 17	951 ± 310	18.25	78 ± 8	35.77
S14	488933.3	4271469.5	49 ± 1	239 ± 12	17 ± 6	490 ± 104	12.95	52 ± 6	35.09
S15	488936.4	4271469.7	17 ± 4	85 ± 3	5 ± 1	193 ± 14	58.20	27 ± 3	26.10
S16	488938.7	4271470.0	22 ± 3	97 ± 2	6 ± 2	266 ± 23	54.75	46 ± 6	30.75
S17	488941.6	4271469.9	26 ± 2	41 ± 1	2 ± 1	203 ± 16	83.90	101 ± 7	25.32
S18	488944.4	4271469.9	30 ± 4	86 ± 3	15 ± 5	629 ± 97	28.90	28 ± 11	42.09
S19	488930.5	4271472.0	15 ± 3	199 ± 3	62 ± 11	1282 ± 529	42.50	18 ± 3	37.98
S20	488933.4	4271472.4	25 ± 6	157 ± 5	18 ± 8	550 ± 142	23.75	52 ± 5	37.59
S21	488936.3	4271472.5	23 ± 8	177 ± 6	12 ± 3	267 ± 33	20.60	14 ± 3	32.03
S22	488939.1	4271472.1	20 ± 4	123 ± 3	9 ± 2	435 ± 36	55.90	44 ± 5	37.42
S23	488941.9	4271472.6	21 ± 10	161 ± 7	19 ± 6	526 ± 93	25.50	27 ± 3	41.28
S24	488944.9	4271472.7	14 ± 15	118 ± 4	17 ± 11	433 ± 185	17.60	12 ± 3	41.67
S25	488930.6	4271474.9	28 ± 4	166 ± 3	20 ± 7	642 ± 108	41.40	18 ± 3	34.71
S26	488933.7	4271475.1	50 ± 7	532 ± 7	17 ± 5.	590 ± 96	39.60	23 ± 4	29.75
S27	488936.6	4271475.1	21 ± 4	141 ± 3	8 ± 3	405 ± 48	63.10	43 ± 6	35.66
S28	488939.3	4271475.4	30 ± 32	136 ± 8	7 ± 4	236 ± 87	9.25	9 ± 2	42.81
S29	488942.0	4271475.0	25 ± 27	145 ± 8	27 ± 17	415 ± 203	11.15	10 ± 2	45.19
S30	488944.8	4271475.1	39 ± 15	154 ± 9	11 ± 8	276 ± 142	9.90	7 ± 5	43.28
Bay	488831.9	4271535.6	18 ± 2	69 ± 1	7 ± 1	161 ± 12	93.35	5 + 2	40.95

## 2 Experimental design, materials, and methods

This dataset presents the activities of natural radioactive tracers measured in groundwater discharging directly to Indian River Bay, DE, USA—not water sampled from nearby wells or from surface water. This distinction is important, because when sampling wells at the coast, chemical processes that occur during flow through aquifers or mixing with surface water can generate differences between wells and discharging water--a full discussion can be found in Duque et al. [1]. The dataset shows the scale of variability in activity of ^223^Ra, ^224^Ra, ^226^Ra, ^228^Ra, and ^222^Rn in this natural system, and the range of spatial variability that can be detected over short distances—information that is essential for defining end members needed to estimate SGD with radioactive tracers.

The study area was selected for being relatively geologically homogeneous [3]. Aquifer salinity has important implications for Ra mobility [1], so seepage meter locations were selected to capture the fresh/saline transition of the submarine aquifer. Seepage meters were arranged in a 5 × 10 grid (3 m spacing) covering a 180 m^2^ (12 × 27 m) area that was shallow (0.3–1.5 m water depth), bathymetrically approximately flat, and nearshore (within 20 m of the shore line). Inshore of the first seepage meter, a low hydraulic conductivity layer prevents SGD between the shoreline and study area. Each seepage meter was positioned with high precision real time kinematic (RTK) GPS.

Seepage meters were installed one week in advance of sample collection in the field site to allow the flushing of the seepage meter chamber that, based on the fluxes measured, was completed several times in all seepage meters. Each seepage meter was sampled five times collecting water for two hours over a semi-diurnal tidal cycle (9:00–11:00, 11:00–13:00, 13:00–15:00, 15:00–17:00, 17:00–19:00). Empty, labeled bags were used for sample collection. The use of empty bags may have an effect on SGD flux measurements, but this practice avoids contaminating collected samples with prefill water.

Samples for ^222^Rn are sensitive to degassing, which likely occurs in seepage meters, so shallow porewater samples were collected from 25 cm depth (below the seabed) for ^222^Rn analysis. We assumed this shallow porewater was representative of water being discharged from the aquifer. We collected the minimum volume required for the analysis to avoid drawing surface water into the sample. Samples were collected slowly with a minipiezometer (MHE products) and syringe using minimal suction to avoid degassing. Sampled water was immediately stored in 250-mL gas-tight bottles that were filled from the bottom and overflowed prior to capping to minimize degassing and atmospheric exchange of gases. A campaign laboratory was installed near the field area to immediately measure ^222^Rn activities using a RAD7 radon detector with RAD H2O accessory. The analysis protocol was adapted to decrease the uncertainty of the ^222^Rn content of the samples.

For analysis of Ra isotopes, Ra was pre-concentrated by adsorption from each water sample onto 20 g (dry weight) of Mn-oxide coated acrylic fiber which adsorbed Ra from the water in the field site directly after collection by passing the water via gravity feed at < 1L/minute through a cartridge containing the fiber [[4], [5], [6]]. ^223^Ra and ^224^Ra were measured on a Radium Delayed Coincidence Counter (RaDeCC) system using the protocols described by Knee et al. (2008) [7] and Street et al. (2008) [8]. Initial ^223^Ra and ^224^Ra measurements were made within 10 days of collection, and all samples were run again within 3–6 weeks of collection to correct for ^228^Th- supported ^224^Ra activity. The error associated with each short-lived Ra isotope measurement was calculated using methods described by Garcia-Solsona et al. (2008) [9]. For ^226^Ra and ^228^Ra, the Mn-fiber samples were ashed at 700 °C and sealed in polypropylene vials. Two high-purity Ge gamma spectrometers measured sample gamma emissions. Data were normalized to known quantities of certified NIST Ra solutions of ^226^Ra and ^228^Ra adsorbed to 20 g of Mn-oxide coated acrylic fiber. Specific activities and one sigma errors were calculated using standard counting techniques [10].

## References

[1] Duque C., Knee K.L., Russoniello C.J., Sherif M., Abu Risha U.A., Sturchio N.C., Michael H.A. (December 2019). Hydrogeological processes and near shore spatial variability of radium and radon isotopes for the characterization of submarine groundwater discharge. J. Hydrol..

[2] Lee D.R. (1977). A Device for measuring seepage flux in lakes and estuaries. Limnol. Oceanogr..

[3] Russoniello C.J., Fernandez C., Bratton J.F., Banaszak J.F., Krantz D.E., Andres A.S., Konikow L.F., Michael H.A. (2013). Geologic effects on groundwater salinity and discharge into an estuary. J. Hydrol..

[4] Dulaiova H., Burnett W.C. (2004). An efficient method for γ -spectrometric determination of radium-226, 228 via manganese fibers. Limnol Oceanogr. Methods.

[5] Kim G., Burnett W.C., Dulaiova H., Swarzenski P.W., Moore W.S. (2001). Measurement of 224Ra and 226Ra activities in natural waters using a radon-in-air monitor. Environ. Sci. Technol..

[6] Moore W.S., Reid D.F. (1973). Extraction of radium from natural waters using manganese-impregnated acrylic fibers. J. Geophys. Res..

[7] Knee K.L., Layton B.A., Street J.H., Boehm A.B., Paytan A. (2008). Sources of nutrients and fecal indicator bacteria to nearshore waters on the north shore of Kaua'i (Hawai'i, USA). Estuar. Coasts.

[8] Street J.H., Knee K.L., Grossman E.E., Paytan A. (2008). Submarine groundwater discharge and nutrient addition to the coastal zone and coral reefs of leeward Hawai'i. Mar. Chem..

[9] Garcia-Solsona E., Masqué P., Garcia-Orellana J., Rapaglia J., Beck A.J., Cochran J.K., Bokuniewicz H.J., Zaggia L., Collavini F. (2008). Estimating submarine groundwater discharge around Isola La Cura, northern Venice Lagoon (Italy), by using the radium quartet. Mar. Chem..

[10] Mook W.E. (2001). Environmental Isotopes in the hydrological cycle. Princ.Appl..

